# Respiration and the F_1_Fo-ATPase Enhance Survival under Acidic Conditions in *Escherichia coli*

**DOI:** 10.1371/journal.pone.0052577

**Published:** 2012-12-28

**Authors:** Yirong Sun, Toshihiko Fukamachi, Hiromi Saito, Hiroshi Kobayashi

**Affiliations:** 1 Guangzhou Institutes of Biomedicine and Health, Chinese Academy of Sciences, Guangzhou, Guangdong, People’s Republic of China; 2 Interscience Co. Ltd, Tokyo, Japan; 3 Faculty of Pharmaceutical Sciences, Teikyo Heisei University, Chiba, Japan; 4 Graduate School of Pharmaceutical Sciences, Chiba University, Chiba, Japan; Dowling College, United States of America

## Abstract

Besides amino acid decarboxylation, the ADP biosynthetic pathway was reported to enhance survival under extremely acidic conditions in *Escherichia coli* (Sun *et al.,* J. Bacteriol. 193∶ 3072–3077, 2011). *E. coli* has two pathways for ATP synthesis from ADP: glycolysis and oxidative phosphorylation. We found in this study that the deletion of the F_1_Fo-ATPase, which catalyzes the synthesis of ATP from ADP and inorganic phosphate using the electro-chemical gradient of protons generated by respiration in *E. coli*, decreased the survival at pH 2.5. A mutant deficient in *hemA* encoding the glutamyl tRNA reductase, which synthesizes glutamate 1-semialdehyde also showed the decreased survival of *E. coli* at pH 2.5. Glutamate 1-semialdehyde is a precursor of heme synthesis that is an essential component of the respiratory chain. The ATP content decreased rapidly at pH 2.5 in these mutants as compared with that of their parent strain. The internal pH was lowered by the deletion of these genes at pH 2.5. These results suggest that respiration and the F_1_Fo-ATPase are still working at pH 2.5 to enhance the survival under such extremely acidic conditions.

## Introduction

*Escherichia coli* has to pass through the extremely acidic stomach before entering the more hospitable gastro-intestinal tract, and hence resistance to extremely acidic environments (AR) is an important mechanism for *E. coli* to survive [1], [2]. Multiple metabolic pathways have been reported to function for survival under extremely acidic conditions. Three amino acid-dependent systems have been identified as enhancing the AR in *E. coli* [1]. The most potent system is the glutamate-dependent system (AR2) [3], [4]. The other two systems are arginine-dependent (AR3) [5], [6] and lysine-dependent (AR4) [7]–[9] systems. Recently an adenosine-dependent AR system was reported in *E. coli*, and this system was less active than AR2 but more potent than AR4 [10]. These systems were proposed to regulate the intracellular pH (pHi) at a higher level than the pH of the surroundings [1], [10].

Why is such pHi regulation required for survival at acidic pH? The most likely explanation may be that some metabolic pathways are required to function for survival under acidic conditions even if cells are unable to grow and that their activity decreases with the decrease in pHi. Our group has reported that the deletion of genes for the metabolic pathway to synthesize ADP was demonstrated to decrease the AR in *E. coli*, suggesting that ATP-dependent metabolic pathways contribute to survive under acidic conditions [11]. Furthermore, it was demonstrated that one such system was the DNA repair system [11].

*E. coli* has two pathways for ATP synthesis from ADP: glycolysis and oxidative phosphorylation. F_1_Fo-ATPase catalyzes the synthesis of ATP from ADP and inorganic phosphate using the electro-chemical gradient of protons generated by respiration in oxidative phosphorylation. In addition to ATP synthesis, the respiratory chain has been reported to regulate pHi in *E. coli* [12], and F_1_Fo-ATPase was shown to regulate pHi in other bacteria [13]. In the present study, we found that both respiration and the F_1_Fo-ATPase function at pH 2.5 to enhance the AR in *E. coli*.

## Materials and Methods

### Bacterial Strains and Culture Media

The bacterial strains used in this study are listed in Table 1. *E. coli* was grown at 37°C in 4 to 10 ml of minimal E medium [17] containing 0.4% glucose (designated EG). The medium pH was adjusted by the addition of NaOH to 7.5 and 7.0 or HCl to 5.5 and 2.5. LB (Luria-Bertani broth) and LB containing 0.4% glucose (designated LBG) media were also used as a rich medium. Antibiotics were used as the following concentrations: tetracyclin, 10 µg/ml; kanamycin, 25 µg/ml. Delta-aminolevulinic acid (ALA) was used at 100 µg/ml.

**Table 1:** Bacterial strains and plasmids used in this study.

Strains	Genotype/description	Reference/source
W3110	λ^−^ F^−^ derived from *E. coli* K-12	[14]
BW25113	*lacI* ^q^ *rrnB*_T14_ *ΔlacZ*_WJ16_ *hsdR514 ΔaraBAD*_AH33_ *ΔrhaBAD*_LD78_	[15]
JW3710	BW25113 *atpD*::km^(1)^	Keio collection^(2)^
JW3715	BW25113 *atpE*::km	Keio collection
DK8	HfrPO1 *bglR thi1 relA1 ilv*::Tn*10* Δ*atpBEFHAGDC*	[16]
ME8366	HfrC *glpR glpD hemA30 zch*::Tn*10*	ME collection^(2)^
SE020	W3110 *atpD*::km	This study;W3110xP1(JW3710)
SE021	W3110 *atpD*::km *hemA30 zch*::Tn*10*	This study; SE022xP1(JW3710)^(3)^
SE022	W3110 *hemA30 zch*::Tn*10*	This study;W3110xP1(ME8366)^(3)^
SE023	W3110 *atpE*::km	This study; W3110xP1(JW3715)

1 km is a gene conferring resistance to kanamycin.

2 Keio and ME collections were obtained from the National BioResource Project (National Institute of Genetics, Mishima, Japan): *E. coli*.

3 These strains required ALA for growth in LB medium and the growth cessation in E madium containing 0.4% glycerol instead of glucose was complemented by a plasmid having *hemA*, suggesting the mutation of *hemA*.

### Measurement of the AR

The AR was measured with cells grown in the logarithmic growth phase as described previously [11] with the following modifications. After the cells had been precultured overnight in LB (for the wild type) or LBG (for mutants) with antibiotics in strains resistant to antibiotics, the cells were diluted 500-fold with EG medium at pH 7.5 and cultured at 37°C until the optical density at 600 nm (OD_600_) reached 0.3∼0.4. Two ml of the culture medium were centrifuged at 5,000×g for 5 min, and the cells in the pellet were suspended with 4 ml of EG medium at pH 5.5. The cell suspension was incubated for 4 h without shaking for the acidic adaptation [18], and the adapted cells were challenged in a 40-fold volume of EG medium at pH 2.5. After incubation at 37°C for 1 or 2 h, the cells were diluted with phosphate-buffered saline [11] and plated on LBG agar plates. Colonies appearing after overnight culture at 37°C were counted, and viability was expressed as described previously [11]. The measurement was repeated three times using separate culture, and the mean value and the standard deviation were calculated.

### Measurement of the ATP Content

After the cells had been cultured as described above, the cells were chilled on ice and then centrifuged at 10,000×g for 5 min at 4°C. The pellets were treated with a solution containing 20 mM Tris-HCl, 50 mM MgSO_4_, 4 mM EDTA, and 50% methanol at pH 7.4 for 30 min at 70°C [19] and then were centrifuged at 10,000×g for 5 min at 4°C. The ATP content of the supernatant was measured using a luminometer (Turner Designs, Inc.) as described previously [20]. Luciferase and standard ATP were purchased from Sigma Chemical Co. The measurement was repeated three times using separate culture, and the mean value and the standard deviation were calculated.

### Measurement of Intracellular pH (pHi)

The pHi was determined by the distribution of salicylic acids between outside and inside the cells, as described previously [11], [21]. After the cells had been adapted in EG medium at pH 5.5 for 4 h, the harvested cells were suspended in EG medium at pH 5.5 or 2.5 at approximately 1×10^9^ cells per ml, and [^14^C]salicylic acids (10 µM; 0.2 µCi/ml) was added. After incubation at 37°C for 15 min, 1 ml of the medium was centrifuged at 10,000×g for 5 min through an oil mixture (laurylbromide and liquid paraffin). The radioactivity of the supernatant and the pellet were measured to obtain the indicator concentrations outside and inside cells, respectively. The amount of protein in the pellet was measured, and the radioactivity of the pellet was divided by the internal water content calculated from the protein content of the pellet. The pHi was calculated by the following equation:
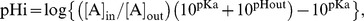
where [A]_in_ and [A]_out_ are the concentrations of salicylic acids inside and outside cells, respectively, and pH_out_ is the medium pH. The pKa of salicylic acids used was 2.89. The measurement was repeated three times using separate culture, and the mean value and the standard deviation were calculated.

### Preparation of the Membrane Fraction

The membrane fraction was prepared as described previously [22], [23] with the following modifications. The cells were cultured in 100 ml of EG medium at pH 7.5 until OD_600_ reached approximately 0.3. The cells were harvested by centrifugation at 5,000×g for 5 min, washed with 0.1 M potassium phosphate buffer at pH 6.6, and resuspended in 5 ml buffer A containing 10 mM Tris-HCl, 140 mM KCl, 2.0 mM β-mercaptoethanol, and 10% glycerol, at pH 8.0. The cells were disrupted through a French pressure cell (Aminco) at 10,000 pounds per inch^2^, and unbroken cells were removed by centrifugation at 10,000×g for 10 min. The membrane fraction was obtained by centrifugation at 100,000×g for 1 h. The obtained membranes were washed once with buffer A and then resuspended with buffer A containing 10% glycerol at 2 to 4 mg protein per ml. All steps were performed at 4°C. The membranes were stored at −20°C until use.

### Measurement of ATPase Activity

The ATP hydrolysis activity in the membranes was determined by the amount of inorganic phosphate (Pi) released from ATP, as previously described [24], [25]. After 5 µg of the membranes had been added to the reaction buffer containing 300 µl of buffer solution (200 mM Tris-maleate and 5 mM MgCl_2_ at pH 9.0) and 270 µl of water, the mixture was incubated at 37°C for 5 min, and then 30 µl of 100 mM ATP was added. After incubation at 37°C for 20 min, the reaction was stopped by the addition 300 µl of cold 15% trichloroacetic acid and immediately chilled on an ice bath. The resulting mixture was centrifuged at 3,000×g for 10 min at 4°C, and 800 µl of the supernatant was mixed with 1.87 ml of the reagent (10 ml of 5 N H_2_SO_4_, 10 ml of 2.5% ammonium molybdate, 10 ml of the solution containing 3% NaHSO_3_ and 1% *p*-methylaminophenol sulfate, and 40 ml of H_2_O). The mixture was incubated at 18°C for 10 min, and the absorbance at 660 nm was measured. K_2_HPO_4_ (10 mM) was used as a standard phosphate. One unit of ATPase activity was defined as the activity to release 1 µmol of Pi from ATP for 1 min. The measurement was repeated three times using separate culture, and the mean value and the standard deviation were calculated.

### Measurement of Proton Pumping Activity

The proton pumping activity of the membranes was determined using the quenching of 9-amino-6-chloro-2-methoxyacridine (ACMA) as described previously [26], [27]. The membranes were suspended with the buffer containing 50 mM 3-(*N*-morpholino)propanesulfonic acid (MOPS) and 10 mM MgCl_2_ (pH 7.5) at 60 µg/ml of membrane protein. After 1 µl of 0.1% ACMA was added to the reaction mixture (1 ml), 5 µl of 200 mM ATP was added. The fluorescence intensity from ACMA was measured with excitation and emission wavelengths of 410 nm and 490 nm, respectively.

### Measurement of the Membrane Permeability to Protons

The membrane permeability to protons was measured as described previously [2], [28] with the following modifications. The *E. coli* cells cultured overnight in LBG medium were diluted 1000-fold with EG medium of pH 7.5 and then were grown at 37°C until OD_600_ reached 0.3∼0.4. The cells were resuspended in the same volume of EG medium at pH 5.5, and cultured for 4 h at 37°C for the acidic adaptation. The adapted cells were collected by centrifugation at 10,000×g for 5 min at room temperature, washed with H_2_O and resuspended in 3 ml H_2_O containing 20 mM MgCl_2_ at 5×10^9^ cells per ml. The measurement was carried out at 25°C with stirring. After 20 µl of 0.2 M HCl had been added, the change of pH was measured with a pH meter connected to a computer. The membrane permeability to protons was represented as one pH unit change per min per mg protein [25]. The measurement was repeated three times using separate culture, and the mean value and the standard deviation were calculated.

### Western Blot Analysis of ATPase Subunits in the Membranes

Western blot analysis of the membrane fraction was carried out as described previously [29], [30] using rabbit antiserum against F_1_ part of *E. coli* F_1_Fo-ATPase which was donated by M. Futai (School of Pharmacy, Iwate medical University, Iwate, Japan). The protein content in the membrane fraction was quantified as described below. Two µg of membrane proteins were mixed with 4×SDS-PAGE sample buffer (125 mM Tris–HCl, pH 6.8, 20% glycerol, 4% SDS, 10% β-mercaptoethanol, and 0.05% bromophenol blue), boiled for 90 seconds, and then applied to a 10% polyacrylamide gel containing 0.1% SDS. Proteins separated by the gel electrophoresis were transferred to a PVDF membrane at 50 volt/cm for 60–70 min. After the PVDF membrane had been incubated with PBS (137 mM NaCl, 2.7 mM KCl, 4.3 mM Na_2_HPO_4_, and 1.4 mM KH_2_PO_4_, pH 7.4) containing 3% BSA for blocking, the membrane was overlaid with 1 ml of antibody diluent solution (3 mM Tris-HCl buffer containing 45 mM NaCl, 3% BSA, and 10% FBS, pH 7.6) containing 1 µl of antiserum against F_1_ part of *E. coli* F_1_Fo-ATPase. The membrane was washed 2 times with TBS-Tween (10 mM Tris-HCl buffer containing 150 mM NaCl and 0.1% Tween 20, pH 7.6) and overlaid with 1 ml of antibody diluent containing 5 µl of anti-rabbit antibodies conjugated with alkaline phosphatase (Biosource, USA). After the membrane was washed 2 times by TBS-Tween, staining was carried out as described previously [29.30].

### Other Methods

Transduction with P1*kc* was performed as described previously [31]. Protein was measured as described previously [32], and bovine serum albumin was used as a standard.

## Results

### Enzyme Activities in the F_1_Fo-ATPase Mutants

Oxidative phosphorylation is mediated by the respiratory chain and the F_1_Fo-ATPase in *E. coli* [33]. *E. coli* F_1_Fo-ATPase consists of two parts, F_1_ and Fo, which contain five subunits (α, β, γ, δ, and ε) and three subunits (a, b, and c), respectively [34], [35]. We used mutants deficient in *atpD* (SE020) and *atpE* (SE023) in this study. *atpD* and *atpE* encode the β and c subunits, respectively [36]. We also used DK8 [16] in which all genes for the F_1_Fo-ATPase were deleted.

**Figure 1:**
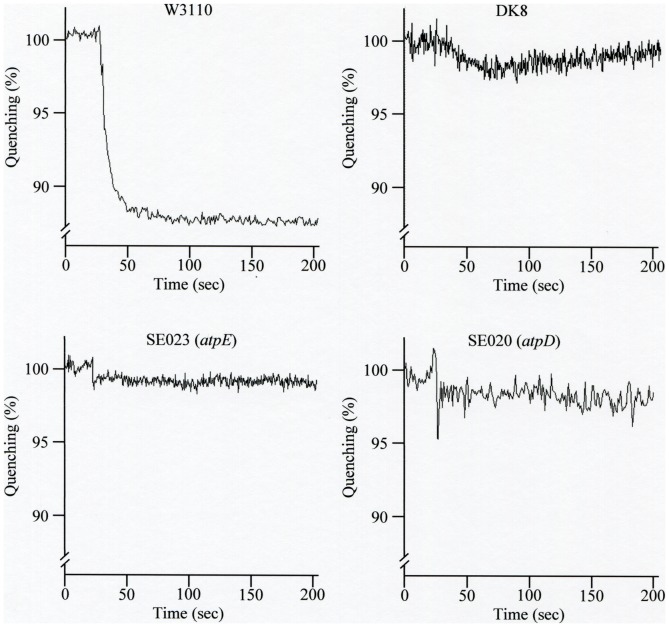
Proton pumping activity of the mutants and the wild type strain. W3110 (wild type, parent strain of SE mutants), DK8, SE023 (*atpE*), and SE020 (*atpD*) were grown, and proton pumping activity was measured as described in Materials and Methods. ATP (1 mM) was added at zero time.

Since the optimum pH of the ATP hydrolysis activity was 9.0 [24], the ATP hydrolysis activity was measured at pH 9.0. The activity was 0.52±0.17 µmol Pi/min/mg protein in the wild type, while the activity was not detected (less than 0.01 µmol Pi/min/mg protein) in any of the F_1_Fo-ATPase mutants at pH 9.0. The proton pumping activity was impaired in these mutants (Fig. 1). The wild type strain grew in the E medium containing lactic acid instead of glucose at pH 5.5, but none of the F_1_Fo-ATPase mutants grew under this condition, indicating that the oxidative phosphorylation was still active at pH 5.5 in the wild type strain but not in the F_1_Fo-ATPase mutants. These results suggested that the F_1_Fo-ATPase activity was negligible in these mutants.

### The AR of Mutants Deficient in a Gene for the F_1_Fo-ATPase or Heme Synthesis

**Figure 2:**
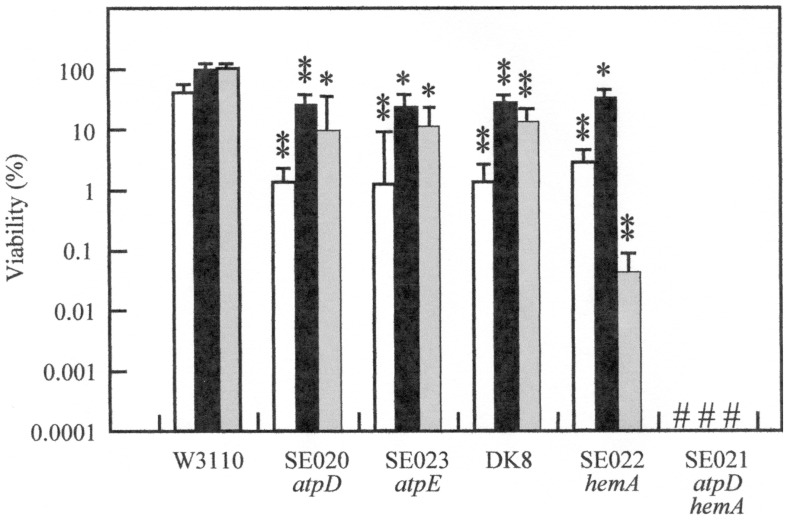
The survival of various mutants after 1 h challenge at pH 2.5. After W3110 (wild type, parent strain of SE mutants), SE020 (*atpD*), SE023 (*atpE*), DK8, SE022 (*hemA*), and SE021 (*atpD hemA*) had been grown in EG medium at pH 7.5 until OD_600_ reached 0.3 to 0.4, the cells were adapted for 4 h at pH 5.5 and challenged for 1 h at pH 2.5 as described in Materials and Methods. SE022 (*hemA*) and SE021 (*atpD hemA*) were precultured overnight with the addition of ALA (100 µg/ml) and then diluted with EG medium at pH7.5 without ALA. ALA was not added to media of pH 5.5 and 2.5. Data from three independent experiments are expressed as mean ± S. D. Symbols: white bars, no addition; black bars, 0.1 mM glutamate was added to media of pH 5.5 and 2.5; gray bars, 0.1 mM arginine was added to media of pH 5.5 and 2.5; #, survival rate was too low to detect (less than 0.001%). The average values and standard deviations obtained from three experiments using separate cultures are represented. One asterisk, p<0.01 compared with the wild type; two asterisks, p<0.005 compared with the wild type.

The stationary-phase cells may be resistant to various stresses [4]. To minimize the responses to stresses other than acidic stress, cells growing logarithmically were used in the present study. F_1_Fo-ATPase mutants SE020, SE023 and DK8 showed AR of *E. coli* decreased about 20-fold compared with that of the wild type W3110 after the cells were challenged at pH 2.5 for 1 h (Fig. 2), and none of these mutants survived after 2 h challenge at pH 2.5 (data not shown). The mutant deficient in *hemA* encoding glutamyl tRNA reductase (SE022) had a low ability to survive at pH 2.5. Glutamyl tRNA reductase is the enzyme that synthesizes glutamate 1-semialdehyde in *E. coli* [37], [38]. Delta-aminolevulinic acid (ALA) is a precursor of heme biosynthesis and is synthesized from glutamate 1-semialdehyde. Therefore, ALA was added to produce heme in the *hemA* mutant as indicated.

When glutamate or arginine was added, the survival of the ATPase mutants was increased, but the survival rate was still lower than that of the wild type strain (Fig. 2). The addition of glutamate increased the survival of the *hemA* mutant, but surprisingly the addition of arginine decreased survival in the *hemA* mutant (Fig. 2). The reason for this decrease is still unknown. We next constructed a double mutant deficient in both *atpD* and *hemA*. After the double mutant had been cultured overnight in LBG with 100 µg/ml ALA, the cells were transferred to the EG medium at pH 7.5 and then to pH 5.5 medium without the addition of ALA. Although the double mutant could grow in the medium at both 7.5 and 5.5 at a slower rate than that of the single mutant, the double mutant could not survive after 1 h challenge at pH 2.5. Even if glutamate or arginine was added, the survival of the double mutant was very low (less than 0.0001%, Fig. 2). These results suggest that either respiration, or the F_1_Fo-ATPase, is essential for survival at pH 2.5 in *E. coli* since both could not be eliminated simultaneously.

### ATP Content of the Mutants Deficient in the F_1_Fo-ATPase and Heme Protein

**Figure 3:**
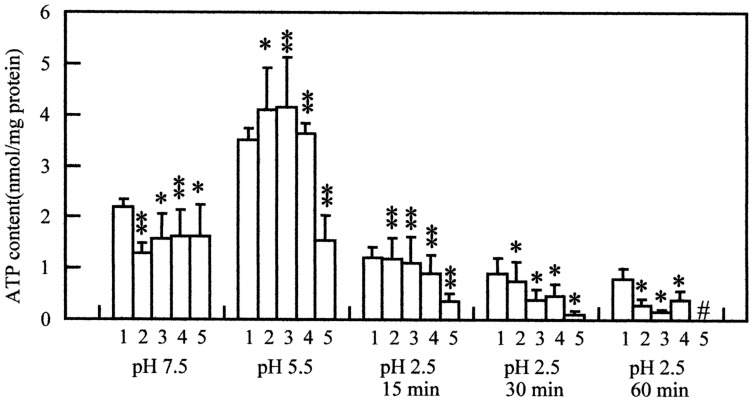
ATP content of various mutants. DK8, SE020 (*atpD*), SE023 (*atpE*), SE022 (*hemA*), and W3110 (wild type, parent strain of SE mutants) were cultured as described in the legend of Fig. 2, and the ATP content was measured as described in Materials and Methods. Strains: 1, W3110 (wild type); 2, SE020 (*atpD*); 3, SE023 (*atpE*); 4, DK8; 5, SE022 (*hemA*). Data from three independent experiments are expressed as mean ± S. D. #, the ATP content was less than 0.01 nmol/mg protein. The average values and standard deviations obtained from three experiments using separate cultures are represented. One asterisk, p<0.01 compared with the wild type; two asterisks, p<0.005 compared with the wild type.

In order to examine whether the ATPase mutants and the respiratory chain mutant affect the ATP content, we investigated the ATP content in the mutants. The ATP content was decreased at pH 7.5 in the F_1_Fo-ATPase mutants, but not at pH 5.5 (Fig. 3). In contrast, the ATP content of the *hemA* mutant was lower than that of its parent strain at pH 5.5 (Fig. 3). These data indicated that the ATP synthetic activity of glycolysis is enough to compensate the ATP consumption at pH 5.5 but the activity of oxidative phosphorylation is not. The ATP content of these mutants decreased more rapidly at pH 2.5 than that of the wild type strain, and the decrease was more rapid in the *hemA* mutant than that in the F_1_Fo-ATPase mutants (Fig. 3). The ATP content in the *hemA* mutant was lower at pH 5.5 and decreased more rapidly at pH 2.5 as compared with that of the *purA* and *purB* mutants reported previously [11], although the survival was almost the same between the *hemA* and *purB* mutants after the acidic challenge at pH 2.5 for 1 h (data not shown). The survival of the *hemA* mutant was significantly lower than that of the *purB* mutant after 2 h challenge at pH 2.5 (data not shown). The ATP content of the double mutant deficient in *atpD* and *hemA* at pH 5.5 was less than 0.01 nmol per mg protein. These data support the previous result that ATP content is an important factor for survival of *E. coli* in acidic conditions [11].

### Effect of Acidic pH on the Expression of the F_1_Fo-ATPase

**Figure 4:**
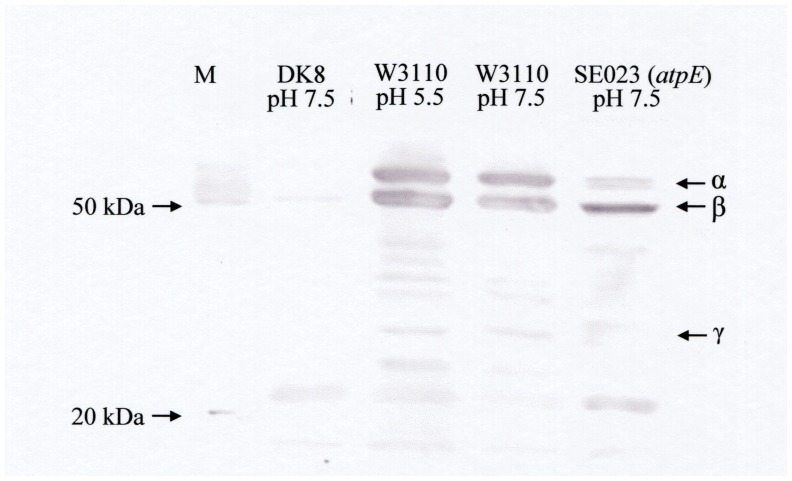
Expression of the F_1_Fo-ATPase. DK8, W3110 (wild type), and SE023 (*atpE*) were grown at the pH indicated, and the amounts of F_1_ subunits were measured with Western blot analysis as described in Materials and Methods. M, molecular weight marker.

The F_1_ portion of the ATPase is not composed of integral membrane proteins and is associated with the membrane-imbedded Fo subunits. The expression of the F_1_ part of the F_1_Fo-ATPase in the membranes was investigated with Western blot analysis. The results implied that the expression of the F_1_ subunits was not affected significantly by the pH change (Fig. 4), ruling out the possibility that the elevated ATP content at pH 5.5 was due to the increase in the amount of the ATPase. The amount of the F_1_ α subunit was decreased in the *atpE* mutant that is deficient in the Fo c subunit (Fig. 4), indicating that proper assembly of the holoenzyme was impaired in this strain.

### Intracellular pH (pHi) in the Mutants Deficient in the F_1_Fo-ATPase and Heme Protein

The pHi values of all of the F_1_Fo-ATPase mutants used in this study were lower than that of the wild type strain (Table 2). The pHi of the *hemA* mutant was also low, but higher than that of the F_1_Fo-ATPase mutants (Table 2). These data indicated that the F_1_Fo-ATPase and the respiratory chain were important for pHi regulation.

**Table 2:** Intracellular pH in various mutants.

	pHo			
strains	5.5	2.5		
		15 min	30 min	60 min
W3110	7.16±0.09	4.08±0.03	3.94±0.04	3.82±0.04
DK8	6.98±0.20**	3.69±0.04**	3.54±0.07**	3.50±0.10**
SE020 (*atpD*)	7.04±0.14*	3.61±0.04**	3.57±0.13**	3.54±0.12**
SE023 (*atpE*)	7.13±0.15*	3.56±0.21**	3.58±0.18**	3.54±0.20**
SE022 (*hemA*)	7.15±0.06	3.79±0.03**	3.71±0.07**	3.64±0.02*

pHi was measured as described in Materials and Methods. pHo is the pH values of the medium. The p-values compared with the wild type were calculated. * p<0.05 (n = 6); ** p<0.005 (n = 6).

We measured the membrane permeability to protons as described previously [2], [28]. The initial velocities of pH change after acid pulse were 0.022±0.009 and 0.021±0.007 pH (n = 3) per min per mg protein in the wild type W3110 and DK8, respectively, in the pH range from 4.1 to 4.7. Similar results were obtained with the *atpD* and *atpE* mutants (data not shown). The *hemA* mutant showed similar permeability (0.022±0.006 pH per min per mg protein, n = 3). These data indicated that the decreased pHi in the mutants was not due to an increase in the membrane permeability to protons.

## Discussion

Multiple metabolic pathways may be required for survival of *E. coli* under extremely acidic conditions [1], [39]. Our group reported that adenosine deamination increased survival under extremely acidic conditions, in addition to amino acid decarboxylation [10]. Furthermore, our group implied that ATP is required for survival under acidic conditions and that one of the ATP-dependent systems is a DNA repair system in *E. coli* [11]. It was found in the previous study that the deletion of *purA* and *purB*, genes for purine biosynthesis, and the gene for ADP synthesis from AMP decreased the ATP content and the AR in *E. coli* [11]. In the present study, we investigated the effect of the deletion of genes required for ATP synthesis from ADP on the AR. Both mutants deficient in the genes for the F_1_Fo-ATPase and the biosynthesis of heme showed rapid decreases in ATP content and low survival at pH 2.5. The F_1_Fo-ATPase consists of two parts, F_1_ and Fo, which contain five and three subunits, respectively [34]. Mutants deficient in *atpD* and *atpE* were used in the present study. *atpD* and *atpE* encode the β subunit of F_1_ and the c subunit of Fo, respectively [36]. The mutants deficient in other subunit genes showed similar results (data not shown). We also used DK8, in which all genes for the F_1_Fo subunits are deleted, and the *hemA* mutant. The present data obtained with these mutants suggested that the F_1_Fo-ATPase and respiration and each contribute to high survival under extremely acidic conditions.

It has been proposed that pHi regulation is an indispensable factor for AR [1], [10]. The pHi was low in both mutants deficient in the F_1_Fo-ATPase and heme proteins. Our present data suggested that the membrane permeability to protons was not impaired by the deletion of these enzymes. It has been argued that respiration has an essential role in pHi regulation in *E. coli* [27]. Consistent with this hypothesis, the pHi regulation was impaired in the *hemA* mutant (SE022). The pHi regulation was also impaired in the F_1_Fo-ATPase mutants even if the respiration was working suggesting an additional level of control. Two possibilities can be argued. The first one is that the F_1_Fo-ATPase extrudes protons to regulate pHi instead of the ATP synthesis at acidic pH. Such a function of the F_1_Fo-ATPase was first demonstrated in *E. hirae* (formerly *S. faecalis*) [13], and was also argued for in *E. coli* [1], although there has been no direct evidence to suggest it *in E. coli*. The second one is that *E. coli* has an unidentified system for pHi regulation whose operation is driven by ATP. The activity of this putative system is diminished by a decrease in the ATP level. The pHi was still regulated at a higher level in the medium even though no ATP was detected in the *hemA* mutant after the acidic challenge for 1 h (Table 2, and Fig. 3). This supports that ATP-independent systems such as amino acid decarboxylation operate to regulate pHi. The addition of glutamate and arginine could increase the viability of the F_1_Fo-ATPase mutants, but the survival was still lower than that of the wild type (Fig. 2), indicating that the amino acid systems alone are not sufficient for AR.

### Conclusions

Intracellular pH affects the enzyme activity, protein stability, structure of nucleic acids, and functions of many other biological molecules. We found in the present study that respiration and the F_1_Fo-ATPase participate in pHi regulation and maintenance of the ATP content at a high level to enhance the AR of *E. coli*. Since pHi regulation is important for survival at acidic pH, *E. coli* is likely to have multiple systems for pHi regulation. In any case, it was strongly suggested that the ATP-dependent metabolic processes enhance the survival at acidic pH even if growth stops and that pHi regulation is indispensable to keep such metabolic processes active.
